# The nutritional equation: decoding diet’s influence on breast cancer risk and progression – a perspective

**DOI:** 10.1097/MS9.0000000000003612

**Published:** 2025-07-18

**Authors:** Emmanuel Ifeanyi Obeagu

**Affiliations:** Department of Biomedical and Laboratory Science, Africa University, Mutare, Zimbabwe

**Keywords:** breast cancer, carcinogenesis, dietary patterns, nutraceuticals, nutrition

## Abstract

Breast cancer is the most commonly diagnosed cancer among women worldwide, with rising incidence influenced not only by genetic and hormonal factors but also by lifestyle determinants, particularly diet. Mounting evidence indicates that nutrition plays a significant role in both the risk and progression of breast cancer through mechanisms involving hormonal modulation, inflammation, oxidative stress, and immune regulation. As dietary patterns can either mitigate or exacerbate oncogenic pathways, understanding the nutritional impact on breast carcinogenesis is essential for developing preventive and adjunctive therapeutic strategies. Specific nutrients and dietary components have shown variable effects on breast cancer development. Diets high in saturated fats, processed foods, and alcohol have been consistently associated with increased risk, while consumption of fiber, omega-3 fatty acids, antioxidants, and plant-based foods appears protective. Additionally, bioactive compounds such as phytochemicals, probiotics, and nutraceuticals like curcumin and resveratrol have demonstrated promising anti-cancer properties by influencing key molecular pathways involved in tumor growth and immune modulation.

## Introduction

Breast cancer remains a major global health concern, representing the most frequently diagnosed cancer and a leading cause of cancer-related mortality among women^[1,2]^. According to the World Health Organization, more than 2.3 million women were diagnosed with breast cancer in 2020, and nearly 685 000 succumbed to the disease^[3,4]^. While significant advancements have been made in early detection, targeted therapy, and survivorship care, the growing incidence highlights the critical need to address modifiable risk factors to prevent disease onset and progression. Among the array of modifiable lifestyle factors, diet and nutrition have emerged as particularly influential in the pathogenesis and trajectory of breast cancer. Accumulating epidemiological and experimental evidence suggests that dietary patterns, individual nutrients, and specific bioactive compounds can significantly influence breast cancer risk, tumor biology, and patient outcomes. These dietary influences may occur through various biological mechanisms including hormone regulation, inflammation control, oxidative stress reduction, and alterations in the gut microbiome. Notably, population-based studies reveal a striking disparity in breast cancer incidence and outcomes across different regions, with lifestyle – particularly dietary behavior – playing a substantial role. For example, Western dietary patterns characterized by high consumption of processed foods, red meats, and saturated fats are linked to higher breast cancer risk, whereas traditional diets like the Mediterranean diet, rich in fruits, vegetables, and healthy fats, are associated with a protective effect. This observation underscores the value of diet as a potentially powerful, non-invasive tool for cancer prevention^[5–9]^.

In addition to its role in cancer initiation, nutrition also affects tumor progression and treatment response. Dietary components can modulate metabolic and signaling pathways that regulate cell proliferation, apoptosis, angiogenesis, and metastasis. Furthermore, nutritional status influences treatment tolerance, immune function, and quality of life in breast cancer patients. Malnutrition, obesity, and metabolic syndrome have all been associated with poorer prognosis and increased recurrence risk. Recent advances in molecular nutrition and nutrigenomics provide deeper insight into how specific nutrients interact with genetic and epigenetic factors to influence individual cancer susceptibility and therapeutic outcomes. For example, omega-3 fatty acids, phytoestrogens, and antioxidants have demonstrated the ability to alter gene expression, regulate inflammatory cytokines, and affect estrogen receptor activity. These findings highlight the emerging potential of personalized nutrition as a component of precision oncology (Table 1)^[10–14]^.

**Table 1:** Dietary components and their influence on breast cancer risk and progression

Dietary component	Food sources	Proposed effect	Mechanism of action
Saturated and trans fats	Red meat, processed foods, fried snacks	↑ Increased risk	Promote inflammation, estrogen synthesis
Omega-3 fatty acids	Fatty fish, flaxseeds, walnuts	↓ Reduced risk	Anti-inflammatory, inhibit tumor cell proliferation
High glycemic carbohydrates	White bread, sugary drinks, pastries	↑ Increased risk	Raise insulin and IGF-1 levels, promoting mitogenesis
Whole grains and fiber	Brown rice, oats, legumes	↓ Reduced risk	Improve insulin sensitivity, lower estrogen levels
Animal protein	Red meat, processed meats	↑ Increased risk	Linked to carcinogenic compounds and oxidative stress
Plant-based protein	Lentils, tofu, beans	↓ Reduced risk	Contains antioxidants and anti-cancer phytochemicals
Vitamin D	Fortified foods, sunlight, fatty fish	↓ Protective effect	Promotes cell differentiation and immune modulation
Folate	Leafy greens, legumes, fortified cereals	↕ Dual effect (dose-dependent)	Supports DNA repair, excess may promote proliferation
Phytoestrogens	Soy, flaxseed, legumes	↕ Protective or risk-enhancing	Mimic estrogen; effect varies by receptor status
Antioxidants	Berries, nuts, green tea, vegetables	↓ Protective effect	Neutralize free radicals, reduce DNA damage
Obesity & caloric excess	Excess caloric intake, poor dietary habits	↑ Increased progression and recurrence	Adipose tissue promotes inflammation and estrogen production

## Aim

The aim of this review is to critically examine the role of diet and nutrition in breast cancer risk, progression, and treatment outcomes.

## Dietary patterns and breast cancer risk

The relationship between overall dietary patterns and breast cancer risk has garnered increasing attention as researchers move beyond studying isolated nutrients to considering the combined effects of entire diets. Dietary patterns represent habitual food consumption behaviors that can influence metabolic and hormonal environments, inflammation, and oxidative stress, all of which are critical in the pathogenesis of breast cancer. Instead of evaluating individual food items or nutrients in isolation, the assessment of dietary patterns provides a more holistic understanding of how diet contributes to cancer development, particularly in the context of long-term exposures and synergistic effects of foods^[14,15]^. One of the most extensively studied dietary patterns in relation to breast cancer is the Western diet, characterized by high intakes of red and processed meats, refined grains, saturated fats, sugary desserts, and low consumption of fruits, vegetables, and whole grains. Numerous epidemiological studies have demonstrated that adherence to this dietary pattern is associated with increased breast cancer risk, especially among postmenopausal women. The Western diet is thought to promote carcinogenesis through various mechanisms, including obesity-related inflammation, insulin resistance, and elevated levels of circulating estrogens and insulin-like growth factors. These factors collectively create a pro-tumorigenic environment that facilitates the initiation and progression of breast malignancies^[16,17]^.

In contrast, the Mediterranean diet has been consistently associated with a protective effect against breast cancer. This dietary pattern emphasizes the intake of fruits, vegetables, legumes, whole grains, nuts, olive oil, and moderate consumption of fish and wine, with limited intake of red meat and processed foods. Rich in antioxidants, polyphenols, and healthy monounsaturated fats, the Mediterranean diet has been shown to exert anti-inflammatory and anti-proliferative effects. Clinical and observational studies suggest that women who adhere to this pattern have a lower risk of breast cancer incidence and mortality. Moreover, the Mediterranean diet is associated with reduced estrogen levels and improved insulin sensitivity, which are favorable in mitigating hormone-dependent breast cancer risks. The plant-based diet, which includes vegetarian and vegan dietary approaches, also shows potential in reducing breast cancer risk. These diets tend to be high in dietary fiber, phytochemicals, and antioxidants while being low in saturated fat. Fiber, in particular, has been shown to reduce circulating estrogen levels by enhancing fecal excretion and altering gut microbiota, thereby lowering the estrogenic stimulation of breast tissue. In addition, phytochemicals such as flavonoids, lignans, and isoflavones present in soy and other plant-based foods may exert weak estrogenic or anti-estrogenic effects, influencing the risk of hormone-receptor-positive breast cancer^[14,15]^.

Another dietary pattern under investigation is the Dietary Approaches to Stop Hypertension (DASH) diet, which is rich in fruits, vegetables, low-fat dairy, whole grains, and lean protein sources. While initially designed for blood pressure control, the DASH diet shares similarities with other anti-inflammatory dietary patterns and has shown potential in reducing the risk of several chronic diseases, including certain cancers. Emerging evidence suggests that adherence to the DASH diet may be inversely associated with breast cancer risk, possibly due to its role in reducing obesity, oxidative stress, and systemic inflammation. Low-carbohydrate and ketogenic diets, although popular in weight management and metabolic control, present a more complex relationship with breast cancer. Some preclinical studies indicate that ketogenic diets, by reducing glucose availability and insulin signaling, may slow the growth of certain breast cancer cell lines. However, clinical evidence is limited, and concerns remain regarding the long-term safety and sustainability of these diets. More robust, controlled trials are needed to clarify their role in breast cancer prevention or management. Cultural and regional dietary patterns also influence breast cancer risk. Traditional Asian diets, which are rich in soy products, green tea, and fish, have been associated with lower breast cancer incidence compared to Western diets. Soy isoflavones, in particular, have received attention due to their selective estrogen receptor-modulating properties. While early concerns about soy potentially stimulating estrogen-receptor-positive tumors existed, current consensus suggests that moderate soy intake is safe and may even offer protective effects, especially when consumed regularly from a young age^[16,17]^.

## Specific nutrients and breast cancer modulation

While dietary patterns offer a broad perspective on cancer prevention, focusing on specific nutrients enables a deeper understanding of the molecular and cellular mechanisms by which diet influences breast cancer risk and progression. Certain macro- and micronutrients have been shown to exert either protective or promotive effects on breast tissue, depending on their role in metabolic regulation, oxidative balance, hormonal modulation, and gene expression. Fats and fatty acids are among the most extensively studied nutrients in relation to breast cancer. Saturated fats, primarily found in animal products and processed foods, have been linked to increased breast cancer risk, particularly among postmenopausal women. These fats may promote tumor development by increasing circulating estrogen levels and enhancing inflammation. In contrast, omega-3 polyunsaturated fatty acids (PUFAs) – abundant in fatty fish like salmon, mackerel, and sardines – are associated with anti-inflammatory properties and may reduce breast cancer cell proliferation and angiogenesis^[18,19]^. Experimental studies have demonstrated that omega-3 PUFAs can modulate cell membrane composition, inhibit NF-κB signaling, and induce apoptosis in malignant cells, highlighting their potential as chemopreventive agents. Carbohydrates and dietary fiber also play a crucial role in breast cancer modulation. High glycemic index and glycemic load diets are associated with increased risk due to repeated insulin spikes, which can enhance insulin-like growth factor 1 (IGF-1) signaling – a known mitogen for breast epithelial cells. Conversely, dietary fiber, particularly from whole grains, legumes, fruits, and vegetables, has been shown to reduce breast cancer risk. Fiber facilitates the excretion of estrogen via feces and supports beneficial gut microbiota, which in turn may influence systemic inflammation and estrogen metabolism. Studies have consistently shown that high fiber intake is inversely associated with estrogen-receptor-positive breast cancer, making it a valuable component in risk reduction strategies^[20,21]^.

Vitamins and antioxidants have garnered significant attention for their potential in counteracting oxidative damage – a hallmark of cancer development. Vitamin D, in particular, has emerged as a critical modulator of breast cancer risk and prognosis. Epidemiological data indicate that low serum vitamin D levels are associated with increased breast cancer incidence and poorer outcomes. Mechanistically, vitamin D regulates cell proliferation, differentiation, and apoptosis through the vitamin D receptor (VDR) present in breast tissue. Clinical trials have also suggested that adequate vitamin D levels may improve response to therapy and reduce recurrence rates, although further research is needed to establish optimal dosing and timing. Similarly, vitamin A (retinoids) and vitamin E (tocopherols) possess antioxidant properties that may protect DNA from damage and inhibit malignant transformation. Retinoids have been shown to regulate gene transcription involved in cell growth and differentiation, while vitamin E may reduce oxidative stress and enhance immune function. However, clinical trials on antioxidant supplementation have yielded mixed results, with some studies suggesting that high-dose antioxidant use during chemotherapy may interfere with treatment efficacy. Thus, while naturally occurring antioxidants in foods are generally beneficial, caution is warranted with supplemental use during active cancer treatment^[22–25]^.

Phytoestrogens, plant-derived compounds that mimic or modulate estrogen activity, have been studied extensively for their dualistic role in breast cancer. Isoflavones, found predominantly in soy products, exhibit selective estrogen receptor modulator (SERM)-like effects, which means they can exert estrogenic or anti-estrogenic effects depending on the hormonal environment. In premenopausal women, isoflavones may compete with endogenous estrogens for receptor binding, thereby exerting a protective effect. However, in estrogen-deprived postmenopausal tissue, their weak estrogenic activity has raised concerns. Nevertheless, the majority of data suggests that moderate consumption of whole soy foods does not increase breast cancer risk and may actually be beneficial, especially in Asian populations where lifelong soy intake is common. Minerals such as selenium, zinc, and calcium have also been implicated in breast cancer modulation. Selenium, a trace element with antioxidant properties, is involved in the activity of glutathione peroxidase and other selenoproteins that protect against oxidative damage. Low selenium levels have been associated with increased breast cancer risk in some populations, although results are inconsistent. Zinc plays a role in DNA synthesis, repair, and immune function, while calcium has been proposed to inhibit cell proliferation and promote differentiation. Despite these potential roles, the evidence for mineral supplementation is less conclusive than for vitamins and requires further investigation through well-controlled trials^[26–29]^.

## Bioactive food compounds and nutraceuticals in breast cancer

The interface between nutrition and oncology has uncovered the therapeutic potential of bioactive food compounds and nutraceuticals in the prevention and management of breast cancer. These naturally occurring chemical constituents, found in fruits, vegetables, spices, grains, and other dietary sources, possess properties that go beyond basic nutrition. They influence cellular and molecular pathways that underlie carcinogenesis, making them promising agents in both cancer prevention and adjunctive therapy. Nutraceuticals – products derived from food sources that offer medical or health benefits – have also attracted significant attention for their role in modulating inflammation, oxidative stress, angiogenesis, and hormone regulation in breast cancer contexts^[21,30]^. Among the most extensively studied bioactive compounds are polyphenols, including flavonoids, phenolic acids, lignans, and stilbenes. These compounds, abundant in tea, berries, red wine, and soy, exhibit antioxidant, anti-inflammatory, and antiproliferative effects. Epigallocatechin gallate (EGCG) from green tea has been shown to suppress estrogen receptor signaling, induce apoptosis, and inhibit angiogenesis in breast cancer cells. Similarly, resveratrol, a stilbene found in grapes and red wine, exerts anti-tumor effects through modulation of p53, PI3K/Akt, and NF-κB pathways, influencing cell cycle arrest and apoptosis. Lignans – present in flaxseeds and whole grains – can modulate estrogen metabolism, thereby offering potential protection against hormone-receptor-positive breast cancers^[31,32]^.

Carotenoids, such as beta-carotene, lycopene, and lutein, are another class of bioactive compounds known for their antioxidative capacity. Found in colorful vegetables like carrots, tomatoes, and leafy greens, carotenoids scavenge reactive oxygen species, mitigating DNA damage and supporting immune defense. Lycopene, in particular, has demonstrated inhibitory effects on the proliferation of ER-positive and triple-negative breast cancer cells in vitro, suggesting it may influence tumor growth and cell differentiation. Observational studies have also reported inverse associations between circulating carotenoid levels and breast cancer risk, reinforcing the value of a carotenoid-rich diet. Spices and herbs also harbor potent bioactive agents. Curcumin, the active compound in turmeric, has shown anti-inflammatory, antioxidant, and anti-metastatic properties across various breast cancer models. It interferes with key molecular targets such as STAT3, COX-2, and Bcl-2, and downregulates pathways involved in proliferation and metastasis. Although its therapeutic promise is well-documented, curcumin’s clinical application is hindered by low bioavailability – prompting ongoing efforts to develop enhanced delivery formulations such as liposomal curcumin and nanoparticles^[33]^.

Another noteworthy compound is sulforaphane, derived from cruciferous vegetables like broccoli and cabbage. Sulforaphane activates phase II detoxifying enzymes, inhibits histone deacetylases (HDACs), and modulates signaling pathways related to apoptosis and cell cycle control. Preclinical studies suggest its efficacy in suppressing tumor growth and sensitizing cancer cells to chemotherapeutic agents. Alongside sulforaphane, indole-3-carbinol (I3C) from the same vegetable group alters estrogen metabolism favorably, decreasing the formation of potentially carcinogenic metabolites and promoting hormonal balance. In the context of nutraceuticals, omega-3 fatty acids, primarily eicosapentaenoic acid (EPA) and docosahexaenoic acid (DHA), offer anti-inflammatory and anti-proliferative benefits. Sourced from fatty fish and marine oils, these lipids can modulate inflammatory cascades and have been linked to reduced breast cancer cell viability and invasion in vitro. Additionally, probiotics and prebiotics are gaining traction for their ability to modulate gut microbiota, which in turn may influence systemic estrogen levels, immune surveillance, and inflammatory responses – all of which are relevant to breast cancer biology^[22]^.

## Diet during and after breast cancer treatment

Nutrition plays a critical role throughout the continuum of breast cancer care, not only in modulating risk and progression but also in influencing treatment outcomes, managing side effects, and supporting long-term survivorship. During active treatment – whether chemotherapy, radiation, surgery, or hormonal therapy – patients often face significant nutritional challenges, including altered taste, loss of appetite, gastrointestinal disturbances, fatigue, and unintended weight changes. These symptoms can compromise nutritional intake, affect treatment tolerance, and impact overall quality of life. As such, dietary strategies during treatment are essential for maintaining nutritional adequacy, preserving lean body mass, supporting immune function, and enhancing the effectiveness of medical therapies. During chemotherapy and radiation therapy, individualized nutritional plans are vital to meet the fluctuating metabolic demands and manage side effects. Protein requirements, for example, increase due to tissue repair and immune cell regeneration. Patients are encouraged to consume high-biological-value proteins from sources such as lean meats, legumes, dairy, and soy. Small, frequent meals rich in nutrient-dense foods are often recommended to combat nausea and anorexia. Hydration is equally important, particularly in managing chemotherapy-related toxicities. Micronutrients, including antioxidants such as vitamins C and E, selenium, and zinc, may help reduce oxidative stress induced by treatment; however, their supplementation must be approached with caution. High doses of antioxidants during therapy have raised concerns about potential interference with the pro-oxidant mechanisms by which chemotherapy and radiation exert their effects. Therefore, natural sources of antioxidants from fruits and vegetables are preferred over pharmacological supplementation during active treatment^[34–36]^.

After the completion of primary treatment, dietary focus shifts toward recovery, risk reduction of recurrence, and management of long-term effects. Evidence suggests that breast cancer survivors who adopt a healthy dietary pattern – rich in vegetables, whole grains, fruits, and legumes and low in saturated fats, processed meats, and refined sugars – experience improved overall survival and quality of life. The American Cancer Society and World Cancer Research Fund recommend a plant-based diet with an emphasis on whole foods and limited alcohol intake. Weight management is particularly crucial in survivorship, as obesity is associated with a higher risk of recurrence and mortality, especially in estrogen receptor-positive breast cancer. Thus, survivors are encouraged to engage in regular physical activity alongside dietary modifications to maintain a healthy body weight and reduce metabolic dysfunction^[37–39]^. In addition to physical benefits, dietary practices also influence psychological well-being. Many survivors experience anxiety about recurrence and find empowerment in taking proactive steps through nutrition. However, misinformation and the proliferation of unregulated “anti-cancer diets” pose risks of restrictive eating and nutrient deficiencies. Health professionals should therefore provide evidence-based guidance and consider the cultural, economic, and personal preferences of each patient when designing post-treatment dietary plans. Registered dietitians trained in oncology nutrition can play a key role in delivering tailored advice and dispelling myths. Emerging areas of interest include the role of gut microbiota, anti-inflammatory diets, intermittent fasting, and metabolic interventions in survivorship. Preliminary studies suggest that dietary fiber and probiotic-rich foods may promote a healthy gut microbiome, which in turn could influence systemic inflammation and immune regulation. Intermittent fasting and time-restricted feeding are being investigated for their potential to enhance metabolic flexibility and reduce insulin resistance, though more research is needed before widespread recommendations can be made. Furthermore, nutrigenomics and personalized nutrition based on genetic and metabolic profiling may offer promising future directions for individualized survivorship care^[40–42]^.

## Molecular mechanisms linking diet to breast cancer

Diet modulates a multitude of biological processes including gene expression, hormone metabolism, inflammation, oxidative stress, and immune surveillance – each of which contributes to breast cancer initiation, progression, or suppression. The complexity of these interactions is underscored by the fact that food-derived compounds can act through multiple, overlapping pathways that influence cell proliferation, apoptosis, angiogenesis, and metastasis. One key mechanism involves the modulation of estrogen signaling pathways, which are central to the development of hormone-receptor-positive breast cancer. Certain dietary patterns – particularly those high in animal fat and low in fiber – can increase circulating estrogen levels by influencing hepatic metabolism and enterohepatic recycling. Conversely, diets rich in lignans (from flaxseeds, whole grains, and vegetables) and isoflavones (from soy) contain phytoestrogens that can weakly bind estrogen receptors (ERs), particularly ER-β, exerting anti-estrogenic effects. These compounds may competitively inhibit the binding of endogenous estrogens to ER-α, thereby modulating transcriptional activity, reducing cell proliferation, and promoting apoptosis in hormone-sensitive tissues. Another significant molecular pathway affected by diet is oxidative stress and redox signaling. Reactive oxygen species (ROS) play dual roles in cancer biology: at moderate levels, they promote cellular proliferation and survival; at high levels, they cause DNA damage, genomic instability, and oncogene activation. Antioxidant-rich foods – such as berries, leafy greens, and citrus fruits – contain bioactive compounds like vitamin C, vitamin E, polyphenols, and carotenoids that scavenge ROS and upregulate endogenous antioxidant defenses via the nuclear factor erythroid 2–related factor 2 (Nrf2) pathway. By mitigating oxidative DNA damage, these nutrients help preserve genomic integrity and reduce mutation rates associated with breast cancer^[10,43]^.

Inflammation represents another crucial link between diet and tumorigenesis. Chronic low-grade inflammation promotes an environment conducive to carcinogenesis through the secretion of cytokines (e.g., IL-6, TNF-α), activation of nuclear factor-kappa B (NF-κB), and stimulation of angiogenesis and tissue remodeling. Diets high in saturated fats, refined sugars, and processed meats are known to exacerbate systemic inflammation. In contrast, anti-inflammatory diets – such as the Mediterranean diet, rich in omega-3 fatty acids, fiber, and polyphenols – attenuate inflammatory signaling. Omega-3 polyunsaturated fatty acids (PUFAs), for instance, downregulate pro-inflammatory eicosanoids and modulate gene expression via peroxisome proliferator-activated receptors (PPARs), contributing to tumor suppression. Epigenetic modifications are another emerging area through which diet exerts influence. Nutrients such as folate, vitamin B12, choline, and betaine are involved in one-carbon metabolism, which regulates DNA methylation – a key epigenetic mechanism governing gene expression. Aberrant DNA methylation patterns are common in breast cancer and can result in the silencing of tumor suppressor genes or the activation of oncogenes. Dietary intake of methyl donors and cofactors can influence these methylation patterns, potentially modifying breast cancer risk. Additionally, certain bioactive compounds like genistein (from soy) and sulforaphane (from cruciferous vegetables) have been shown to inhibit histone deacetylases (HDACs), thereby altering chromatin structure and gene transcription in a manner that favors tumor suppression^[44,45]^.

Insulin resistance and metabolic signaling also link diet to breast cancer risk, particularly in postmenopausal women. Diets high in glycemic load and low in dietary fiber contribute to hyperinsulinemia, which can stimulate the insulin-like growth factor-1 (IGF-1) axis – a potent mitogenic and anti-apoptotic signaling pathway. Elevated IGF-1 levels activate the PI3K/Akt/mTOR pathway, promoting cellular proliferation and survival in breast tissue. Dietary interventions that enhance insulin sensitivity – such as increased fiber intake, reduced refined carbohydrate consumption, and weight loss – can modulate this pathway, potentially reducing cancer risk and progression. Studies highlight the role of the gut microbiome as a mediator of diet-cancer interactions. The composition and diversity of gut bacteria are shaped by dietary habits and, in turn, influence systemic estrogen levels, immune responses, and metabolite production. For instance, a fiber-rich diet supports the growth of beneficial microbiota that produce short-chain fatty acids (SCFAs) such as butyrate, which possess anti-inflammatory and anti-proliferative properties. Furthermore, certain gut bacteria can metabolize dietary polyphenols and phytoestrogens into bioactive forms that interact with breast cancer–related pathways^[46–49]^.

## Conclusion

The interplay between diet and breast cancer represents a critical dimension in both the prevention and management of this complex disease. A growing body of epidemiological, clinical, and molecular evidence supports the role of dietary patterns, specific nutrients, bioactive compounds, and nutraceuticals in modulating breast cancer risk and progression. Diet influences key biological pathways, including hormone regulation, oxidative stress, inflammation, epigenetic modification, and metabolic signaling – each of which can drive or suppress carcinogenesis. Plant-based diets rich in fruits, vegetables, whole grains, and healthy fats, particularly those following Mediterranean or anti-inflammatory principles, have shown protective associations, while Western-style diets high in processed foods and saturated fats are linked to increased risk. Moreover, bioactive food compounds such as polyphenols, carotenoids, flavonoids, and omega-3 fatty acids exhibit promising anti-tumor properties by targeting molecular pathways involved in proliferation, angiogenesis, and apoptosis. These findings open avenues for integrating dietary strategies into personalized breast cancer prevention and adjunctive care. However, challenges such as interindividual variability, bioavailability of nutraceuticals, and the complexity of diet-cancer interactions underscore the need for more rigorous, long-term, and mechanistically grounded research.

## Data Availability

Not applicable as this a perspective.

## Research registration unique identifying number (UIN)

Not applicable as this a perspective.
